# Disparities and barriers in the assessment of psychological distress, access to and use of psycho-oncological support in Europe: current perspectives

**DOI:** 10.3389/fpsyg.2023.1252843

**Published:** 2023-09-19

**Authors:** Veronica Coppini, Giulia Ferraris, Dario Monzani, Roberto Grasso, Gabriella Pravettoni

**Affiliations:** ^1^Applied Research Division for Cognitive and Psychological Science, IEO, European Institute of Oncology IRCCS, Milan, Italy; ^2^Department of Psychology, Educational Science and Human Movement (SPPEFF), University of Palermo, Palermo, Italy; ^3^Department of Oncology and Hemato-Oncology, University of Milan, Milan, Italy

**Keywords:** psycho-oncology, psychological support, psychological distress, cancer disparities, cancer inequalities, cancer divide

## Abstract

The implementation of psycho-oncological support has shown important results in positively influencing treatment outcomes and quality of life in cancer patients and survivors. In the last few decades, the importance of mental health has been brought to attention to the general public and healthcare professionals on a national, institutional and organisational level. Official guidelines, policies, and training programs have been developed suggesting that psycho-oncological support should be considered as a non-negotiable requirement for quality cancer care in many hospitals and clinical centres across Europe. Health organisations, associations, institutions, and societies, such as the International Psycho-Oncology Society (IPOS) and the European Partnership for Action Against Cancer (EPAAC), are forming alliances, funding research projects and organising congresses in order to study, understand, and discuss the reasons for barriers and disparities in psycho-oncological support and, eventually, to overcome the existing cancer divide. Nevertheless, the World Health Organization’s (WHO) estimations indicate that the cancer burden is still increasing, and relevant barriers and disparities in accessing psycho-oncological support continue to exist and influence the health conditions and quality of life of cancer patients and survivors. The present work will present the current disparities and barriers regarding assessment, access to and use of psycho-oncological support in the countries of the European Union, making suggestions for further research and possible solutions.

## 1. Introduction

Over the past few decades, the world’s scientific community has started to inquire more attentively into psychological and social matters arising in the oncological context. The resulting literature highlighted the emotional responses of cancer patients and their families as well as the social and behavioural factors influencing cancer mortality and morbidity (Holland, 2018). The latter issues underlined the need for an interdisciplinary approach that includes oncological, psychiatric, and psychological care and, thus, promoted the evolving of a new discipline, Psycho-Oncology, aimed at supporting oncological patients on the psychosocial, psychobiological and ethical level.

Cancer patients and their loved ones, represent a high-risk population due to the challenges of the disease which, in turn, might provoke feelings of fear and worry, emotional distress, and mental health disorders. In fact, one in three cancer survivors suffers from at least one clinically relevant mental health complication requiring professional psychological support, and there is a higher prevalence of mental disorders in cancer patients, when compared to the general population, across several tumour types (Zimmermann-Schlegel et al., 2017). This represents an important issue by itself, however, the situation appears to be even more critical when considering that emotional distress and psychiatric morbidity in cancer patients are associated with a consistent reduction in quality of life, severe impairment in social relationships, longer rehabilitation time, lower compliance to treatment and care, and shorter survival (Grassi, 2020). Therefore, providing psycho-oncological support is of crucial importance, as a matter of fact, a wide portion of the existing literature indicates its effectiveness in reducing anxiety and depressive symptoms, ameliorating adherence to treatment and, consequently, treatment outcomes, and improving the quality of life of cancer patients, survivors, and their families (Shennan et al., 2011; Greer et al., 2012; Faller et al., 2016).

Cancer societies and institutions such as IPOS and EPAAC, have proposed a Standard of Quality Cancer Care endorsed by scientific bodies and other stakeholders, such as the World Psychiatric Association (WPA) and the Union for International Cancer Control (UICC), highlighting the necessity to recognise psychosocial cancer support as a universal human right. Indeed, for quality cancer care, the psychosocial domain must be integrated into routine cancer care starting from the measurement of emotional distress (Grassi, 2020), as was outlined in the most recent guidelines of the European Society for Medical Oncology (ESMO) (Grassi et al., 2023). Currently, the provision of psycho-oncological support is mandatory in cancer centres certified by the German Cancer Society (GCS) across European countries, however, the implementation quantity and quality of such support widely vary between different centres (Breidenbach et al., 2022). Certainly, one of the staples required from the GCS is patient-centred healthcare and communication in cancer care (German Cancer Society, 2023). However, the individual patient is often reduced to a molecular profile (Carrera and Ormond, 2015); the real challenge in the oncological field would be finding ways to treat both the disease and the patient, leaving space for the patients’ needs and wishes and the provision of detailed information on one’s condition to promote informed decision making (Giordano et al., 2020). In spite of the numerous advances, and the awareness progress regarding the relevance and the need for a structured psycho-oncological program for the implementation of individualised psychological support to cancer patients and their families, there are still several disparities between and within European countries and between centres on a national and regional level, as well as multiple barriers that prevent patients from seeking and obtaining the support and the help they need. Therefore, the aim of the current paper is to illustrate the existing disparities in and barriers to access and use of psycho-oncological support on different levels, which will be presented below. In conclusion, suggestions for improvement and further research will be addressed.

## 2. Disparities

In Europe there are important disparities in the provision of psycho-oncological support in hospitals and cancer centres. Undeniably, disparities in psycho-oncological and general cancer care are rooted in two core factors: firstly, the economic aspect and the allocation of resources, secondly, the level of knowledge and awareness regarding the impact of psychological support on the health and quality of life of cancer patients and their families (Grassi et al., 2016; Holland, 2018). Indeed, among European countries, only 37% have a specific budget for psycho-oncological support and in most eastern European countries mental health is not considered as a priority, on the contrary psychological distress is still stigmatised or its impact underestimated (Grassi et al., 2016; Hook and Bogdanov, 2021). Nonetheless, additional socio-demographic factors, such as age, gender, education, income and residence represent a recurrent reason for disparity in psycho-oncological care. The population with younger age, female gender, better education, greater income, and urban residence receives psycho-oncological support in a higher percentage, and/or quality, when compared to the older, male, less educated, with a lower income population living in rural areas (Zwahlen et al., 2017). Disparities have also been found among different cancer types, indeed, breast cancer patients tend to receive greater provision of psychosocial care than prostate cancer patients; yet, the latter variability may be connected to the gender disparity mentioned above and vice versa (Kowalski et al., 2016).

Concerning cross-countries’ disparities, Eastern and Southern European countries tend to provide worse quality cancer care than Nordic countries. Neamţiu et al. (2016) published a report analysing the availability and provision of psycho-oncological support in national policy documents and in breast cancer care certification schemes across 32 European countries following the recommendations of the EPAAC. As emerged by the above-mentioned report, 25 national and 4 regional cancer plans were identified, with 6 countries (i.e., Bulgaria, Croatia, Iceland, Lichtenstein, Romania, and Slovakia) not reporting any cancer plan, program or strategy regarding the implementation of psycho-oncological support, whilst the remaining 28 countries only mentioned the need for psycho-oncological support. Detailed recommendations and guidelines for psycho-oncological support were reported in only 10 countries’ national plans, including Austria, Belgium, Germany, Netherlands. and Switzerland (Neamţiu et al., 2016). Nonetheless, after 2016, the GCS opened its Cancer Centre Certification Programme to centres in non-German speaking countries in order to improve the quality of general and psychological cancer care within certified networks. As of the last date of consultation of the European Cancer Centres’ website there are 173 certified cancer centres across European countries, the GCS’s certification programme is, in truth, the largest in Europe (European Cancer Centre, 2023).

Although provision of psychosocial support is mandatory in certified centres, the extent and the modalities to which it is dispensed still differ between centres; dissimilarities can be found in screening measurements, national psycho-oncological care quality standards and policies, institutional capacity, training and education programs, and staff availability (Breidenbach et al., 2022). Several disparities exist between and within European countries with regard to the availability, the quality and the quantity of psycho-oncological support in cancer centres. If psycho-oncology support is to be maintained and supported all over Europe, we need to better understand the range of barriers that might contribute to such disparities. Potential barriers that prevent or limit assessment of psychological distress, access to and the use of psycho-oncological support are outlined below.

## 3. Barriers to assessment of psychological distress

As reported in the ESMO guidelines (Grassi et al., 2023), “all patients with cancer should be regularly screened and assessed for anxiety in all phases of illness.” Nevertheless, there are several barriers strongly impacting on the assessment of psychological distress in cancer patients. Firstly, it is necessary to state that the possibility of access to, use, and provision of psycho-oncological support depends directly on the assessment tools [e.g., the Distress Thermometer (National Comprehensive Cancer Network, 2019)], and the Edmonton Symptom Assessment System, ESAS (Bruera et al., 1991) and screening programs of psychological distress that, in turn, are influenced by the awareness of mental health and psycho-oncology. Thus, the lack, or inadequacy, of assessing for psychological distress, often influenced by the organisational, economic, and educational disparities mentioned above, represents a barrier itself. Overall, barriers to assessment of psychological distress in cancer patients can be found on different levels: personal level (e.g., socio-demographic, cultural and psychosocial characteristics, and cancer type), healthcare provider level (e.g., personal beliefs and qualification), and institutional and/or organisational level (e.g., resource allocation, national and international guidelines and standards, training programs, etc.). The three levels are presented below.

### 3.1. Patient level

The literature suggests several racial/ethnic differences in self-reporting psychological distress. For example, Fayanju et al. (2021) found that African-American cancer patients were more likely to report no distress compared to non-Hispanic white patients, despite reporting a similar number of stressors. As a matter of fact, patients had the lowest median score on the Distress Thermometer (National Comprehensive Cancer Network, 2019) with respect to the Asian patients and Hispanic patients population which showed the highest scores. The latter results can be of multiple nature such as religious, cultural or social; indeed, scientific literature often reports on the impact of culture and religion on the stigmatisation of mental health problems and the notion of “normality” regarding emotional distress and unpleasant feelings, which frequently tie back to specific racial/ethnic groups (Andrykowski et al., 2014; Schulze et al., 2022a). Other notable socio-demographic barriers refer to older age, male gender, lower educational level, lower income and ruralness of residence. Rural cancer patients may avoid self-reporting mental distress because of negative attitudes or stigma associated with mental health issues and services that are often recurrent in more isolated geographic areas, where, in addition, the mean income and educational level are generally lower when compared to urban sites (Schulze et al., 2022a). Furthermore, current literature underlines the presence of severe mental illness and/or a comorbid pre-existent mental health disorder as a barrier to being screened for emotional distress and informed about psycho-oncological support services (Günther et al., 2022). Lastly, having a rare cancer type diagnosis could be considered as a barrier. Lung, gynaecological, breast, and gastrointestinal cancer patients are more likely to be screened for distress compared to other types of tumours (Bergerot et al., 2021); however, previous studies in the literature are discordant as they found breast and skin cancer diagnoses as barriers to the assessment of psychological distress (Schulze et al., 2022a).

### 3.2. Healthcare provider level

Healthcare providers have a key role in detecting and assessing psychological distress. Indeed, their attitude, experience, and psychosocial competence might directly affect the use of assessment tools or the investigation for emotional distress. Current literature underlines the positive impact of psychosocial competencies on the implementation of psycho-oncology. On the contrary, the healthcare providers’ negative beliefs regarding psycho-oncology and its value and effectiveness act as a crucial barrier in preventing them from even asking patients if they are experiencing some kind of distress or need for psychological support (Senf et al., 2019). Therefore, there is an impellent need for medical training on psychosocial issues and support from clinic leaders in order to enhance providers’ commitment to psycho-oncology as well as their knowledge on the importance and effectiveness of recommending psycho-oncological support to cancer patients and their families (Frey Nascimento et al., 2019). Another barrier to the assessment of psychological distress in cancer patients, on a healthcare provider level, is the lack of discussing patients at tumour boards; indeed, tumour board discussions have shown to optimise not only somatic treatment, but also the detection of distress in cancer patients (Günther et al., 2021).

### 3.3. Institutional level

There are four main areas that comprise the implementation of psycho-oncological assessment and support on an institutional and organisational level, namely clinical programs, education and training, scientific conferences, and research activities. Regarding clinical programs, many centres and hospitals have staff members who are responsible for the psycho-oncological support, nonetheless only a small number of centres in the world have well developed programs and, more importantly, qualified staff (Holland, 2018). On this note, an important barrier is that of the shortage of qualified providers that are trained and experienced, indeed, several programs depend mainly on volunteers, nurses and clergy members (Holland, 2018). National and international European groups and societies have contributed to the creation of training and educational programs, starting from the European Society for Psychosocial Oncology (ESPO) in 1986, and enhancing clinical teaching and research by promoting conferences, networks and collaborative efforts. In Addition, there are dedicated research groups with the goal of spreading awareness regarding organisational effects on patient-reported processes and outcomes, such as the “Organisational Health Services Research Group” in Germany (Ansmann et al., 2019). However, the lack of hospital personnel, time, and access to dedicated programs represent barriers to the assessment of psychological distress in cancer patients (Bruera et al., 1991). Understandably, financial aspects have to be considered as healthcare costs can be heavy, especially if not covered by insurance or government funds. Financial toxicity can be worsened by the lack of qualified staff which in turn might negatively influence waiting lists and, thus, patients that can afford it are deflected to private care, whilst those who cannot sustain the costs have to wait for long periods of time that often affect the prognosis and their mental health (Bruera et al., 1991; Dee et al., 2021).

## 4. Barriers to access to psycho-oncological support

Access to psycho-oncological support, despite numerous advances regarding accessibility in the last decades, still entails several barriers which are also detected in the reception of general cancer care. Barriers to access are presented below on the three aforementioned levels.

### 4.1. Patient level

Access to psycho-oncological support can be hindered by a variety of personal factors. For instance, ruralness of residence and/or physical impairment represent important barriers to accessing any type of care, however, technology is moving forward and, especially after the Covid-19 pandemic, telehealth has been implemented by various healthcare providers (Schuit et al., 2021; Fischl et al., 2023). Although some patients still prefer face-to-face therapy, televisits may break down the barrier of travelling, isolation, and impairment. In addition, low income and low education might act as barriers both in the access to psycho-oncological support and to devices that would allow the fruition of telehealth, with the risk of accentuating the already persistent cancer divide (Ferraris et al., in press).

### 4.2. Healthcare provider level

Furthermore, there are other relevant barriers to accessing psycho-oncological support, which are related to healthcare professionals and institutions, concerning scarcity of referrals from providers. Indeed, healthcare providers play a crucial role in facilitating access to psycho-oncological support, however, it is reported that they often lack in providing information, that is complete and useful, regarding the location, modalities, specialists and details of the support’s services (Ernst et al., 2018).

### 4.3. Institutional level

At an institutional level, barriers to accessing psycho-oncological support can stem from shortcomings within healthcare organisations. Institutions need to enhance dissemination activities and provide clear directions on their websites to guide patients and their families. Improving awareness and accessibility within healthcare institutions is crucial to ensure that both patients and hospital personnel are informed about the availability of psycho-oncological support services (Ernst et al., 2018).

## 5. Barriers to the use of psycho-oncological support

Clearly, once patients have access to psycho-oncological support, barriers regarding its use may arise. Barriers to use of psycho-oncological support are, again, declined on the three aforementioned levels below.

### 5.1. Patient level

Indeed, there appear to be personal characteristics and perspectives of the patient that can affect their willingness to engage with psycho-oncological support. Aside from socio-demographic aspects (Schulze et al., 2022a), there are recurrent psychosocial aspects, some influenced by socio-demographic ones (such as cultural beliefs and stigma regarding mental health services and problems), that need to be addressed; several patients, for example, report thinking psychological support is not necessary or useful for their perceived level of distress, or that their level of distress is not severe enough to warrant intervention, hence preferring self-help (Clover et al., 2015). In addition, Schulze et al. (2022b) found that high emotional distress (but not physical) acts as a barrier to the utilisation of psycho-oncological support, yet, it appears that only suffering from both emotional and physical distress leads to the wish for support (Schulze et al., 2022b).

### 5.2. Healthcare provider level

Regarding aspects related to healthcare professionals, there seem to be a lack of motivation in providing psycho-oncological support due to extra shortage of personnel, resources, and knowledge on its benefits on cancer patients; as a matter of fact approximately one in six physicians entirely refrains from providing psycho-oncological support (Zimmermann-Schlegel et al., 2017). Furthermore, within the oncology literature, barriers related to the personal discomfort and uneasiness of the healthcare provider in communicating about such sensitive issues with cancer patients and survivors have been detected (Gurren et al., 2022).

### 5.3. Institutional level

At the institutional level, multifaceted barriers contribute to the limited utilisation of psycho-oncological support services. Notably, the physical infrastructure poses challenges, as many healthcare centres lack dedicated spaces for providing these vital services (Luxon, 2015). The absence of suitable environments for confidential discussions can deter patients from seeking or engaging in psycho-oncological support. Furthermore, the temporal aspect of support provision adds complexity (Luxon, 2015). Several centres and hospitals can only offer a brief period of psycho-oncological therapy due to constraints in available resources or structured treatment plans. Once this limited period concludes, patients often face the challenge of being referred to another facility for ongoing support (Schuit et al., 2021). Unfortunately, the referral process is frequently hindered by a lack of clear pathways or bureaucratic complexities, leaving patients uncertain about their next steps. The allocation of physical spaces, together with the extension in the duration of the provided support within the centre, should be crucial points in addressing the existing disparities in psycho-oncological care and integrating it more effectively into the cancer treatment continuum (Greenwood-Lee et al., 2018).

## 6. Discussion

Overall, the main reasons for disparities and barriers appear to be socio-demographic, related to psychosocial aspects, both of the patient and the healthcare professional, economic, and organisational. Therefore, future research should consider investigating each of the aspects related to disparities and barriers, considering various perspectives from different stakeholders. On this note, a team of psychologists, oncologists, data scientists and policymakers from different European countries have cooperated to execute the Beacon Cancer Care Project (BEACON) to map the main capacities and capabilities of cancer centres in Europe with respect to the prevention, diagnosis and treatment of cancer disease. BEACON is a project funded by the European Commission, and it proposes to interview cancer patients, healthcare providers, researchers, and policy makers in the Oncology field across Europe in order to better understand their needs, perspectives, and suggestions on what could be done, and how, so as to reduce disparities and break down barriers at all the levels implicated in the cancer divide (Ferraris et al., under review).^1^ Moreover, precisely in relation to psycho-oncological support, there is a need for national and international guidelines that are updated and more detailed when informing on the procedures and means of psycho-oncological support in hospitals and centres, such as the most recent clinical practice guidelines of the ESMO (European Cancer Centre, 2023). Finally, because of the enormous impact of psychological distress on the health and quality of life of cancer patients and providers, distress should be considered as the “sixth vital sign” and, thus, addressed in accordance (Bultz and Carlson, 2005).

## Data availability statement

The original contributions presented in the study are included in the article/supplementary material, further inquiries can be directed to the corresponding author.

## Author contributions

VC conceptualized the article. VC and GF wrote the first draft of the manuscript. DM, RG, and GP reviewed and edited the manuscript. All authors contributed to the article and approved the submitted version.

## Funding

This work was supported by “Beacon Cancer Care” funded by the European Union’s EU4Health Programme (EU4H) under grant agreement number: 101080005.

## Conflict of interest

The authors declare that the research was conducted in the absence of any commercial or financial relationships that could be construed as a potential conflict of interest.

## Publisher’s note

All claims expressed in this article are solely those of the authors and do not necessarily represent those of their affiliated organizations, or those of the publisher, the editors and the reviewers. Any product that may be evaluated in this article, or claim that may be made by its manufacturer, is not guaranteed or endorsed by the publisher.

## Footnotes

^1^Ferraris, G., Coppini, V., Monzani, D., Grasso, R., Kirac, I., Horgan, D., et al. (under review). Needs and preferences in cancer care: addressing reasons for disparities in the cancer context across Europe. A qualitative study protocol of the Cancer care Beacon project.

## Abbreviations

IPOS: international psycho-oncology society
EPAAC: European partnership for action against cancer
WHO: world health organization
WPA: world psychiatric association
UICC: union for international cancer control
ESMO: European society for medical oncology
GCS: German cancer society
ESPO: European society for psychosocial oncology
BEACON: Beacon cancer care project
